# Development of a clinical-grade ultrasensitive droplet digital PCR test for monitoring of relevant *ESR1* variants by liquid biopsy

**DOI:** 10.1016/j.jlb.2026.100675

**Published:** 2026-10-13

**Authors:** Leisa P. Jackson, Ethan Barnett, Helen Halpin, Brittany D'Alessio, Emma Longshore, Kristen A. Eller, Kathleen Szuhany, Tatiana Erazo, Gary A. Pestano, Pedram Razavi

**Affiliations:** a Biodesix, Inc., 919 W. Dillon Rd, Louisville, CO, 80027, USA; b Memorial Sloan Kettering Cancer Center, 1275 York Avenue, New York, NY, 10065, USA

**Keywords:** Advanced breast cancer, Endocrine resistance, ESR1, Selective estrogen receptor degrader, Liquid biopsy, Digital PCR

## Abstract

Breast cancer is the second most prevalent cancer-type globally. The current standard of care for hormone receptor-positive (HR+), human epidermal growth factor 2-negative (HER2-) advanced breast cancer (ABC) is endocrine therapy (ET) plus cyclin-dependent kinase 4/6 inhibitor (CDK4/6i). Most ABC patients receiving ET + CDK4/6i eventually develop resistance, leading to disease progression. In approximately 20-40% of HR + cases, resistance is driven by acquired activating mutations in the ligand binding domain (LBD) of the estrogen receptor gene (*ESR1*). The next-generation oral selective estrogen receptor degraders (SERDs) comprise an advanced class of ETs intended for use in ER+/HER2- ABC with *ESR1* mutation(s) that has progressed on prior ET. We have developed and validated a New York State Clinical Laboratory Evaluation Program (CLEP)-approved laboratory developed test (LDT) for detection of clinically relevant, and the most prevalent *ESR1* LBD mutations in ABC. The LDT uses advanced multiplexing on the QX600 ddPCR system to identify the presence of *ESR1* E380Q, V422del, S463P, L536 H/P/R, Y537C/D/N/S, and D538G in plasma. Our test was clinically validated for accuracy and found to be 100% concordant when compared to two NGS reference tests (11/11 eligible *ESR1* variants and 18/18 *ESR1* WT samples were correctly called). One variant was detected by the ddPCR test at 0.01% VAF that was not present in the reference dataset, likely due to sample input differences between our test and the reference. Analytic specificity was 100%, and the 95% analytic limit of detection (LoD95) was as low as 0.065% VAF. The Limit of Blank (LOB) was 0 - 3.4 copies depending on the assay. These findings support the use of our multiplexed ddPCR test as a rapid, sensitive, and scalable liquid biopsy approach for identifying emerging *ESR1* mutations and may inform endocrine therapy selection in the treatment of HR+/HER2− advanced breast cancer.

## Graphical abstract

**Figure:**
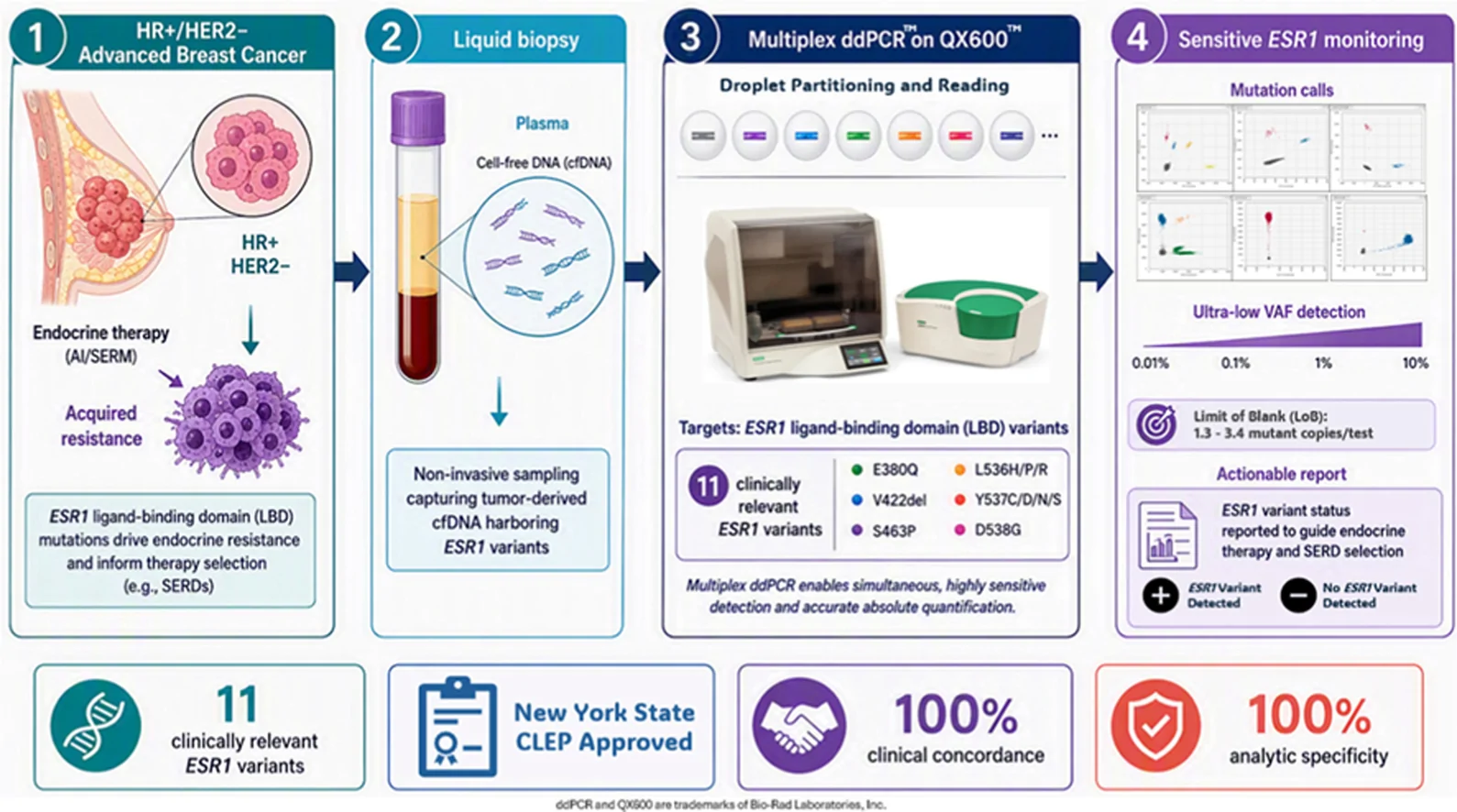

## 1 Introduction

Breast cancer (BC) is one of the leading causes of death among women. In 2024 alone, there were approximately 2.4 million new cases diagnosed and 0.7 million deaths due to BC, the highest number of cancer-related deaths in women worldwide [1]. Hormone receptor-positive (HR+), human epidermal growth factor 2-negative (HER2-) BC is the most common subtype, accounting for approximately 70% of new cases in the US between 2019 and 2022 [2].

The current standard of care for advanced breast cancer (ABC), including locally advanced and metastatic disease, is endocrine therapy (ET) in combination with cyclin-dependent kinase 4/6 inhibitor (CDK4/6i) [3]. Selective estrogen receptor (ER) modulators (SERMs), aromatase inhibitors (AIs), and the first-generation selective estrogen receptor degrader (SERD), fulvestrant, have traditionally been used as the ET backbone in first-line therapies for ABC [4]. AIs block the activity of the aromatase enzyme and prevent conversion of androgens to estrogens, while SERMs and fulvestrant antagonize the ER in breast tissue by directly binding the receptor and inducing a conformational change [5], [6]. Unlike SERMs, fulvestrant acts as a pure antagonist and leads to degradation of the ER [6], [7]. Several studies have shown improved clinical benefits and lower toxicity with AIs than with the SERM, tamoxifen [8]. In the RETRACT survey of US oncologists in early 2024, most clinicians reported treating their first-line HR+/HER2- ABC patients with CDK4/6i + AI. 79% reported that 61-100% of patients received this combination over CDK4/6i + fulvestrant, ET monotherapy, or conventional chemotherapy [9].

Most advanced breast cancer patients receiving ET + CDK4/6i eventually develop resistance, leading to disease progression. In approximately 20-40% of HR + cases, resistance is driven by acquired activating mutations in the estrogen receptor gene (*ESR1*) [10], [11], [12]. These mutations which primarily occur in the ligand binding domain (LBD) of *ESR1* are rarely present in primary tumors (about 1-10%) [11], [13] and cause AI resistance by stabilizing the unliganded ER, allowing for estrogen-independent activity and driving tumor growth [10], [14].

The next-generation oral selective estrogen receptor degraders (SERDs), imlunestrant, elacestrant, giridestrant, and camizestrant, as well as the proteolysis-targeting chimera (PROTAC) ER degrader, vepdegestrant, comprise an advanced class of ETs intended for use in ER+/HER2- ABC that has progressed on prior ET. Similar to fulvestrant, the oral SERDs act by directly antagonizing the estrogen receptor, leading to ER degradation and reduction in ER-modulated signaling. However, they have been shown to degrade ER more effectively, especially in *ESR1*-mutant (*ESR1*m) disease [15]. Based on results from the EMERALD clinical trial, which demonstrated better progression free survival (PFS) with elecastrant as compared to standard of care AI or fulvestrant, ASCO guidelines were updated to recommend *ESR1*m testing from tissue or liquid biopsy (LBx) at recurrence or progression on ET [16]. More recently NCCN and ESMO guidelines were updated to include LBx as the recommended method for *ESR1*m detection in HR+/HER2- ABC [17], [18]. The approved oral SERDs and vepdegestrant are indicated for tumors with *ESR1* mutations, as measured by the Guardant360 LBx-based companion diagnostic (CDx), in patients who have progressed on prior ET/CDK4/6i therapy [19], [20], [21].

The drive to develop effective and safe therapies for the large population of women battling (ET resistant) HR+/HER2- ABC has led to a rapidly evolving therapeutic landscape. Elacestrant, imlunestrant, and vepdegestrant were all approved between 2023 and 2025, and novel approaches continue to emerge. The clinical utility of serial ESR1 mutation monitoring was demonstrated in PADA-1, in which patients with emerging ESR1 mutations detected by ctDNA before radiographic progression were randomized to continue AI + palbociclib or switch to fulvestrant + palbociclib. Early switching significantly improved median PFS (11.9 vs 5.7 months; HR, 0.61; P = 0.004) [22]. Inspired by this proof-of-concept study, in 2025, the Phase III SERENA-6 trial evaluated the clinical utility for another novel strategy: using real-time circulating tumor DNA (ctDNA) monitoring for *ESR1* mutations to guide treatment decisions prior to clinical evidence of progression [23]. In SERENA-6, the next-generation SERD, camizestrant, was shown to improve both first (16.8 vs 9.2 months; HR, 0.45) [23] and second progression free survival (25.7 vs 19.1 months; HR, 0.63) [24] when HR+/HER2- ABC patients were switched from AI plus CDK4/6i to camizestrant plus CDK4/6i in the first line upon detection of *ESR1*-mutated ctDNA. This study highlights the benefit of early intervention based on molecular rather than clinical progression, as well as the need for serial monitoring in the blood via non-invasive, liquid biopsy. At the time of this publication, camizestrant (Etcamah) had just received FDA approval in the United States. Prior to that, it was the first oral SERD granted approval in the European Union for first-line therapy and was previously approved in Japan, Saudi Arabia, and the United Arab Emirates. With camizestrant approval, it is now essential that BC patients are proactively monitored longitudinally for *ESR1* mutations during AI therapy to identify eligible patients for timely initiation of camizestrant treatment.

With the wealth of therapeutic options available to HR+/HER2- ABC patients with *ESR1*-mutated disease, there is a corresponding need for diagnostic tests to complement the current CDx. These tests should be accurate, blood-based, clinical-grade diagnostics; and in the context of the approval of camizestrant capable of detecting mutations at ultra-low concentrations as they emerge. Droplet digital PCR (ddPCR) is a highly accurate, clinically proven testing platform capable of detecting very low concentrations of nucleic acids in high background, making it particularly useful for identifying rare variants [25]. It also presents a lower-cost, higher throughput option versus NGS, more suited for serial testing and monitoring of emergent *ESR1* mutations conferring therapeutic endocrine resistance [26].

Here we report on the development and validation of the GeneStrat *ESR1* ddPCR Test, a new clinical-grade, New York State Clinical Laboratory Evaluation Program (CLEP)-approved laboratory developed test (LDT) for detection of the most prevalent *ESR1* LBD mutations in advanced breast cancer [10], [27]. The LDT uses advanced multiplexing on the QX600 ddPCR system to identify the presence of ESR1 E380Q, V422del, S463P, L536 H/P/R, Y537C/D/N/S, and D538G in liquid biopsy specimens. The validation studies executed to evaluate the performance of the *ESR1* ddPCR test included analytic sensitivity (limit of blank, limit of detection, limit of quantification/linearity), analytic specificity, reproducibility, and clinical accuracy.

## 2 Materials and methods

### 2.1 Donor specimens and cell-free DNA isolation from plasma

Specimens were prospectively collected from adult donors consented under Institutional Review Board (IRB) protocol, and samples were de-identified prior to processing. Cell-free DNA (cfDNA) was prepared as described previously [25]. Briefly, whole blood was collected in cfDNA whole blood collection tubes (Streck) followed by double-spin fractionation to isolate and clarify plasma. cfDNA was extracted from plasma using the QIAamp DSP Circulating Nucleic Acid Kit (Qiagen) and quantified by Qubit dsDNA HS Assay (Thermo Fisher Scientific).

### 2.2 Analytic materials

Analytic specimens were contrived by admixing synthetic double stranded DNA sequences corresponding to the 11 targeted *ESR1* variants (gBlocks, IDT) (Table S1) into a background of wild-type (WT) genomic DNA (gDNA). The WT gDNA had previously been sonicated to generate a cfDNA-like matrix comprised of approximately 150 bp fragments and was also used as an *ESR1*-negative, WT control. The Seraseq ctDNA *ESR1* Mutation Mix AF1% and the Seraseq ctDNA *ESR1* Mix WT were purchased for use from a commercial supplier (LGC SeraCare). The AF1% material is a ctDNA-like mixture of human genomic DNA from the reference cell line, GM24385, and synthetic DNA constructs corresponding to 18 relevant *ESR1* LBD mutations and 4 *PIK3CA* mutations. It was diluted in the ctDNA *ESR1* Mix WT to generate analytic materials at a range of concentrations near the estimated assay limit of detection (LoD).

### 2.3 In-line quality control (QC) materials

The laboratory workflow controls included positive and negative ddPCR controls, as well as positive and negative extraction controls processed with each batch of samples (Table S2). The *ESR1* Analytic Positive Control was designed to contain analyte concentrations at 1-2x the assay LoD.

### 2.4 Droplet digital PCR

Droplet Digital PCR was performed according to the manufacturer's instructions (ddPLEX *ESR1* Mutation Detection Kit and *ESR1* EXV Screening Assay dEXD51651559, Bio-Rad). Each specimen was tested across a minimum of 6 reaction wells, including 2 wells each of the mutant and WT assays as part of the ddPLEX *ESR1* Mutation Detection Kit (“ddPLEX”) and 2 wells of the *ESR1* EXV Screening Assay (“EXV”) (Table 1). The ddPCR Multiplex Supermix (Bio-Rad) was used, and restriction enzymes were omitted from the reaction. The instrument workflow consisted of the Automated Droplet Generator (AutoDG), PTC Tempo Deepwell Thermal Cycler (Bio-Rad), and the QX600 Droplet Reader (Bio-Rad). Data analysis was performed using QXManager software (version 2.3.1 Premium Edition).

**Table 1:** ddPCR Assays Comprising the GeneStrat *ESR1* ddPCR Test. ddPLEX: ddPLEX *ESR1* Mutation Detection Kit; and EXV: *ESR1* EXV Screening Assay.

Assay	*ESR1* Exon #	Mutant Assay Allele	Mutant Assay Channel	Wild Type Assay Allele	Wild Type Assay Channel
ddPLEX	5	E380Q (c.1138G > C)	FAM	380-Wild Type	HEX
	7	S463P (c.1387T > C)	ROX	463-Wild Type	Cy5
	8	L536R (c.1607T > G)	HEX	53X-Wild Type	FAM
		Y537C (c.1610A > G)	Cy5/Cy5.5		
		Y537N (c.1609T > A)	Cy5		
		Y537S (c.1610A > C)	FAM/HEX high		
		D538G (c.1613A > G)	FAM/HEX low		
EXV	6	V422del (c.1265_1267del)	FAM (non-discriminating multiplex)	*ESR1* WT	HEX
	8	L536H (c.1607 T > A)			
		L536P (c.1607 T > C)			
		Y537D (c.1609 T > G)			

## 3 Results

### 3.1 Feasibility

Test feasibility studies were conducted to assess test sensitivity and accuracy using commercially available reference materials and contrived positive specimens. Analyte-positive material was diluted in WT to target 0.025% - 0.2% variant allele frequency (VAF) and 10 ng and 30 ng total input. Accuracy was 100% for detection of all 11 *ESR1* variants at 0.025%-0.05% VAF with at least 2 copies detected at 30 ng input (Fig. 1). At the lower 10 ng input, all 11 variants were detectable at 0.025-0.1% VAF (Fig. S1). No variants were detected in WT-only material or no template controls (Table S3).

**Fig. 1:**
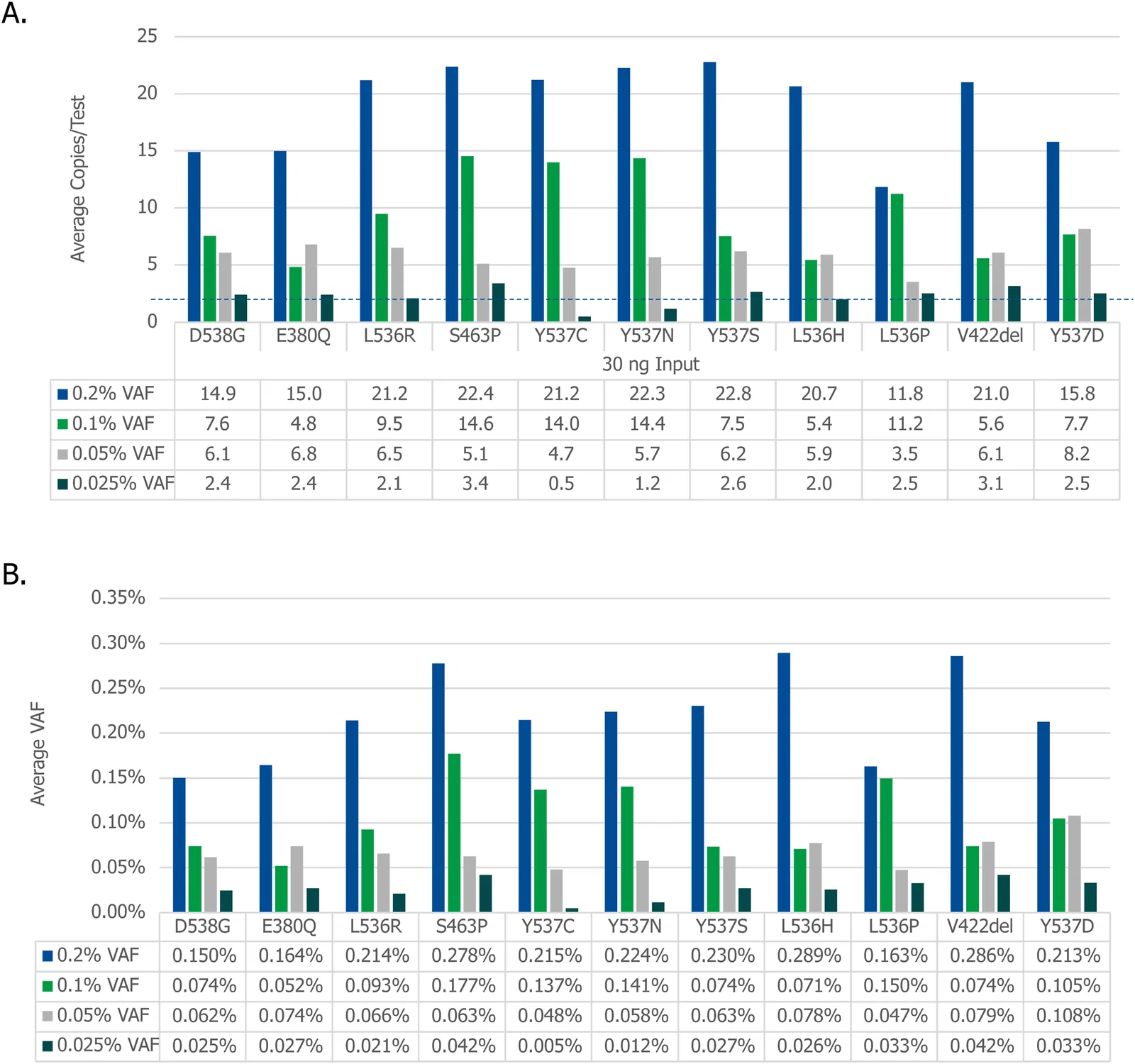
Feasibility. The SeraSeq ctDNA ESR1 Mutation Mix AF1% was used for D538G, E380Q, L536R, S463P, and Y537 C/N/S to assess initial test accuracy. Contrived samples were generated using synthetic dsDNA for L536 H/P, V422del, and Y537D. Samples were tested at 0.2% - 0.025% VAF. The average observed copies per test and the average observed VAF for each concentration are shown in (A) and (B), respectively for the 30 ng input per reaction condition. The analytic threshold for calling a positive was set to 2 copies/test (dotted blue line in (A)).

### 3.2 Limit of blank (LoB) and limit of detection (LoD)

For each study, thirty replicate tests were performed (30 ng cfDNA input per test) using a single lot of the assay across two days. The LoB study was performed using four unique plasma-derived cfDNA samples from NSCLC donors previously confirmed negative for *ESR1* mutations by ddPCR. To assess LoD, four unique low-positive specimens were contrived for each analyte at concentrations near the estimated LoD to generate 30 replicates and processed through the test specimen workflow, starting from nucleic acid isolation. The 95th percentile of blank measurements (LoB95) ranged from 0 to 3.4 mutant copies/test for each of the 11 *ESR1* variants. This resulted in analytic cutoff values for calling a positive result (calculated by rounding up to the nearest integer value from the LoB, but with a minimum value of 2 copies/test) of 2-4 mutant copies/test (Table 2, Table 2a). The limit of detection at 95% confidence (LoD95) ranged from 6.8 to 22.9 mutant copies/test and 0.065% to 0.20% VAF for each of the 11 *ESR1* variants. This resulted in an overall analytic limit of detection for the test (taken as the maximum value observed) of 22.9 mutant copies/test and 0.20% VAF.

**Table 2:** LoB and LoD Summary Table. The LoB, analytic cutoff (for calling a positive vs negative result) and LoD95 are provided. LoD95 is expressed in both mutant copies and VAF with 95% confidence intervals provided. The LoB for variants for V422del, L536H, L536P, and Y537D was assessed at the assay level due to the non-discriminating readout of the EXV assay.

Target	Limit of Blank (Mutant Copies/Test)	Analytic Cutoff (Mutant Copies/Test)	LoD95 (Mutant Copies/Test) [95% CI]	LoD95 (VAF) [95% CI]
D538G	1.3	2	8.7 [0.0, 17.4]	0.082% [0.000%, 0.166%]
E380Q	1.4	2	14.8 [6.0, 23.5]	0.157% [0.061%, 0.253%]
L536R	0	2	8.2 [0.4, 16.0]	0.077% [0.007%, 0.146%]
S463P	1.3	2	9.1 [0.1, 18.1]	0.118% [0.014%, 0.222%]
Y537C	1.4	2	7.9 [0.7, 15.1]	0.074% [0.011%, 0.137%]
Y537N	0	2	6.8 [0.1, 13.5]	0.065% [0.000%, 0.131%]
Y537S	1.3	2	7.5 [0.9, 14.2]	0.071% [0.008%, 0.134%]
V422del	3.4	4	14.3 [5.6, 22.9]	0.149% [0.064%, 0.234%]
L536H			22.9 [12.1, 33.7]	0.201% [0.114%, 0.287%]
L536P			10.9 [3.4, 18.3]	0.104% [0.035%, 0.174%]
Y537D			13.9 [4.9, 22.9]	0.131% [0.040%, 0.222%]

**Table 2:** Normal Healthy Donor (NHD) mutant copy number results. Variant copy number results are provided for n = 10 donor specimens and workflow controls.

Sample	D538G	E380Q	L536R	S463P	Y537C	Y537N	Y537S	L536H/L536P/V422del/Y537D Multiplex
ddPCR Positive Control	99.6	127.6	122.2	101.3	116.5	109.7	99.8	204.9
*ESR1* Analytic Positive Control	17	28.7	21.4	25.9	17	18.6	21.7	56
Negative Specimen Control	0	0	0	0	0	0	0	0
No Template Control	0	0	0	0	0	0	1.4	0
NHD1	1.6	0	0	0	0	0	0	0
NHD2	0	0	0	0	0	0	0	0
NHD3	0	0	0	0	1.3	0	0	0
NHD4	0	0	0	0	0	0	1.4	0
NHD5	0	0	0	0	0	0	0	0
NHD6	0	0	0	0	0	0	0	0
NHD7	0	0	0	0	0	0	0	0
NHD8	1.5	0	0	0	0	0	0	0
NHD9	0	0	0	1.4	0	0	0	0
NHD10	0	0	0	0	0	0	1.3	0

### 3.3 Limit of quantification (LoQ) and linearity

The GeneStrat *ESR1* ddPCR Test is intended for use as a qualitative test in the clinic. We validated the lower limit of quantification as well as the linearity of the test. Inputs were comprised of contrived positive specimens with analyte concentrations spanning the estimated reportable range of the test (approximately 10-1000 mutant copies/test). The LoQ was defined as the most dilute condition where it and all higher concentration conditions resulted in CV ≤ 20% for 3 replicate tests (Table S4) and ranged from 17 to 136 copies/test depending on analyte. The linearity of each assay was assessed across the full range of inputs for each targeted analyte. We performed linear regression analysis comparing the input concentration for each analyte to the measured copy number by ddPCR and found that the coefficient of determination was ≥ 0.95 for all variants (Table S4 and Fig. 2)

**Fig. 2:**
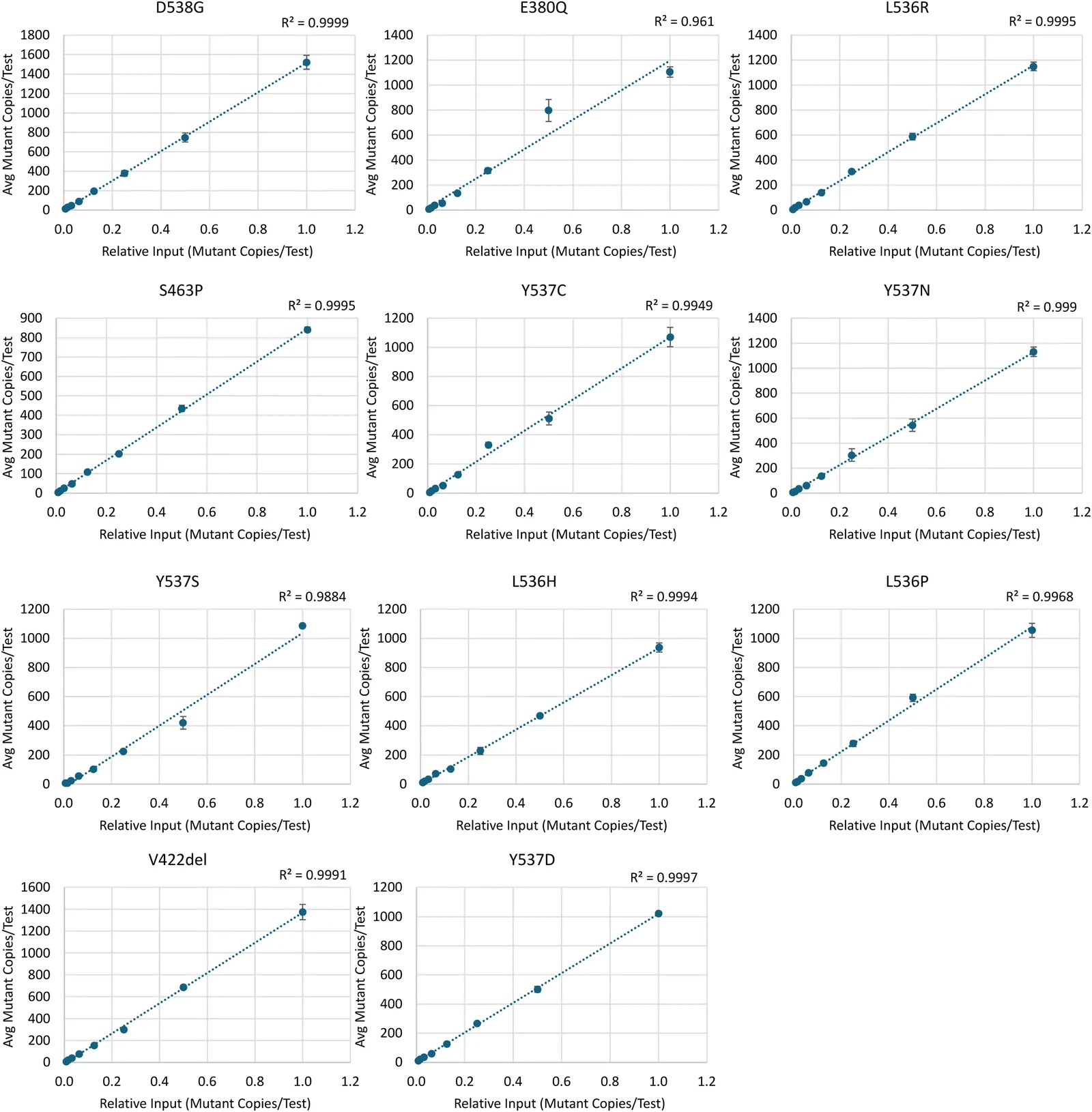
Linearity plots for each of the 11 targeted *ESR1* variants. Trend lines for linear regression analysis and the resulting coefficients of determination (R^2^) are shown. Error bars: standard deviation for 3 replicate tests.

### 3.4 Reproducibility

Five runs were performed across 3 days by 2 operators to assess intra-day, inter-day, and overall reproducibility. Sample inputs included one negative sample and 3 positive samples at high, medium, and low (1-2x LoD levels) mutant copy number in 30 ng total cfDNA per test (Fig. 3). Two different instrument workflows were included in the evaluation. All positive samples tested positive, and all negative samples tested negative. The greatest source of variability overall was run day. The maximum CV observed for all variant and wild-type measures occurred for the low input sample in all cases and was 37% for intra-day reproducibility (3 runs performed on the same day by 2 different operators), 51% for inter-day reproducibility (3 runs performed on 3 separate, consecutive days by 1 operator), and 39% overall (all 5 runs) (Tables S5 and S6) Variability was significantly lower for the higher inputs (targets of 250 and 1000 mutant copies per test), as expected.

**Fig. 3:**
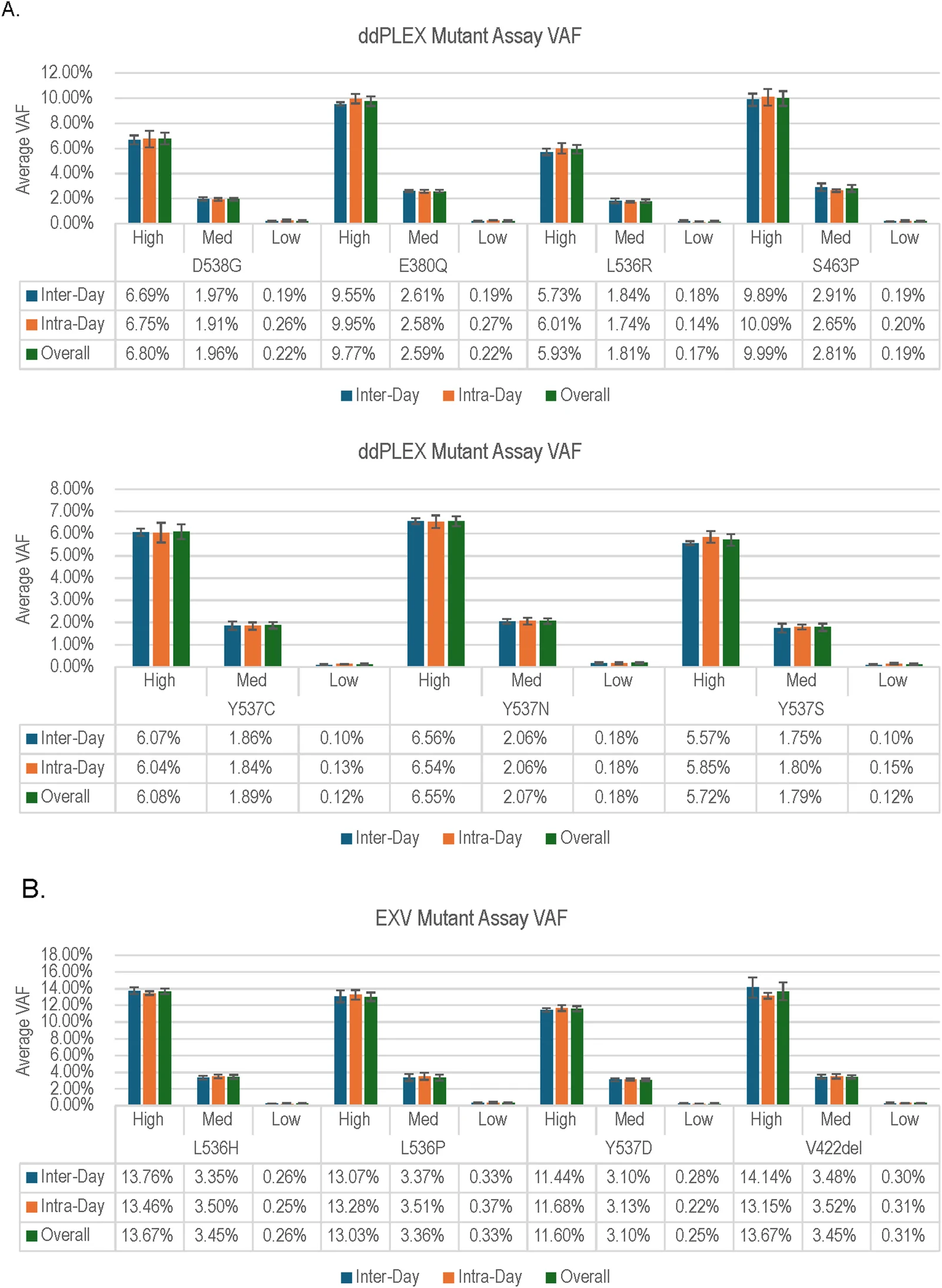
Reproducibility. Average variant allele frequency (VAF) measured for all evaluations (inter-day, intra-day, and overall) are shown for the high, medium, and low (LoD level) positive samples for (A) ddPLEX variants and (B) EXV variants. Error bars: standard deviation for 3 replicate tests for inter- and intra-day assessments and 5 replicates for overall precision.

### 3.5 Specificity

Analytic specificity was performed using whole blood from 10 consented, deidentified presumed-normal donors collected into cfDNA blood collection tubes. The GeneStrat *ESR1* ddPCR Test was 100% specific (Table 2, Table 2b). All ten normal healthy donors tested negative for all 11 *ESR1* variants at inputs ranging from 4 to 10.4 ng cfDNA input per test (mean: 6.5 ng). The measured WT copy numbers are provided in Table S7.

### 3.6 Clinical accuracy

The clinical accuracy cohort consisted of 25 individuals with advanced HR+/HER2-breast cancer with radiographically documented disease progression (per RECIST). Patients receiving genomic profiling utilizing commercially available ctDNA next generation sequencing assays (Guardant360 or MSK-ACCESS) were selected for the study. Guardant360 (55 cancer-associated gene targets), which was used in the SERENA-6 trial to support longitudinal serial ctDNA monitoring of *ESR1* mutations, is the current benchmark for evaluating clinical performance of *ESR1*-mutation monitoring in liquid biopsy [22], [23]. The MSK-ACCESS lab-developed NGS test is used routinely in the clinical at the Memorial Sloan Kettering Cancer Center and assesses 129 cancer-associated gene targets. Both NGS tests include the region of *ESR1*, exon 5-8, that is targeted by the GeneStrat *ESR1* ddPCR test.

Streck cfDNA whole blood (10 – 20 mL) was collected for concurrent clinical profiling (Table S8). cfDNA was isolated from the plasma fraction, and the entire sample was input to the GeneStrat *ESR1* ddPCR Test (mean: 33 ng, IQR: 14-36 ng). The results for the ddPCR test were directly compared to the clinical sequencing results to determine the accuracy of the ddPCR test. Of the 25 specimens evaluated, 18 were *ESR1* WT and 7 tested positive for *ESR1* mutations, including 3 patients who tested positive for multiple *ESR1* mutations (for a total of 11 eligible *ESR1* reference variants) (Table 3). The PPA and NPA between the ddPCR test and the comparative examination were both 100% at the label level (Table 4). The measured variant allele frequencies ranged from 0.01% to 28.7% (Mean: 4.20%, IQR: 0.51%-2.46%) and were highly concordant with reference VAFs (R^2^ = 0.9979) (Fig. 4).

**Table 3:** Clinical Accuracy Study Results. Samples with ddPCR Variant listed as EXV Assay are positive for one or more of the following variants: Y537D, L536H, L536P, V422del. G360: Guardant360; VAF: Variant Allele Frequency; WT: Wild-type.

Specimen ID	Reference Method	Reference Result	Reference VAF	Reference Tumor Fraction	GeneStrat ddPCR Test Result	GeneStrat ddPCR Variant	GeneStrat ddPCR Mutant Copies	GeneStrat ddPCR VAF
MSK-BDX-ESR1-002	G360	V422del	0.30%	5.00%	Positive	EXV Assay	10.1	0.32%
MSK-BDX-ESR1-003	G360	WT	N/A	20.30%	Negative	WT	N/A	N/A
MSK-BDX-ESR1-004	MSK-ACCESS	WT	N/A	Not reported	Negative	WT	N/A	N/A
MSK-BDX-ESR1-006	G360	WT	N/A	0.10%	Negative	WT	N/A	N/A
MSK-BDX-ESR1-009	G360	WT	N/A	<0.05%	Negative	WT	N/A	N/A
MSK-BDX-ESR1-010	G360	WT	N/A	<0.05%	Negative	WT	N/A	N/A
MSK-BDX-ESR1-011	G360	D538G	1.20%	21.10%	Positive	D538G	79.9	1.02%
		Y537N	0.50%		Positive	Y537N	44.7	0.57%
MSK-BDX-ESR1-012	G360	WT	N/A	<0.05%	Negative	WT	N/A	N/A
MSK-BDX-ESR1-013	G360	WT	N/A	<0.05%	Negative	WT	N/A	N/A
MSK-BDX-ESR1-014	G360	WT	N/A	4.70%	Negative	WT	N/A	N/A
MSK-BDX-ESR1-015	G360	D538G	10.30%	60.80%	Positive	D538G	476.8	9.86%
MSK-BDX-ESR1-017	G360	Y537S	0.06%	0.40%	Positive	Y537S	8.4	0.20%
MSK-BDX-ESR1-018	G360	WT	N/A	1.20%	Negative	WT	N/A	N/A
MSK-BDX-ESR1-019	MSK-ACCESS	V422del	1.53%	Not Reported	Positive	EXV Assay	60	1.54%
		Y537C	4.24%		Positive	Y537C	164	5.23%
MSK-BDX-ESR1-021	G360	Y537S	0.70%	1.50%	Positive	Y537S	91	0.70%
MSK-BDX-ESR1-022	G360	WT	N/A	<0.05%	Negative	WT	N/A	N/A
MSK-BDX-ESR1-029	G360	WT	N/A	<0.05%	Negative	WT	N/A	N/A
MSK-BDX-ESR1-031	G360	WT	N/A	0.10%	Negative	WT	N/A	N/A
MSK-BDX-ESR1-033	MSK-ACCESS	WT	WT	Not Reported	Negative	WT	N/A	N/A
MSK-BDX-ESR1-034	MSK-ACCESS	WT	WT	Not Reported	Negative	WT	N/A	N/A
MSK-BDX-ESR1-035	G360	WT	N/A	<0.05%	Negative	WT	N/A	N/A
MSK-BDX-ESR1-037	G360	D538G	30.50%	73.50%	Positive	D538G	8849	28.70%
		N/A	N/A		Positive	L536R	2.95	0.01%
		V422del	0.90%		Positive	EXV Assay	246	0.71%
		Y537S	1.00%		Positive	Y537S	341	1.53%
MSK-BDX-ESR1-038	G360	WT	N/A	1.40%	Negative	WT	N/A	N/A
MSK-BDX-ESR1-039	MSK-ACCESS	WT	WT	Not Reported	Negative	WT	N/A	N/A
MSK-BDX-ESR1-040	G360	WT	WT	0.06%	Negative	WT	N/A	N/A

**Table 4:** Clinical accuracy for the GeneStrat *ESR1* ddPCR test versus the reference NGS tests shown in a sample label-level confusion matrix.

	Reference NGS Result		
		Positive	Negative
**ddPCR**	Positive	7	0
	Negative	0	18

**Fig. 4:**
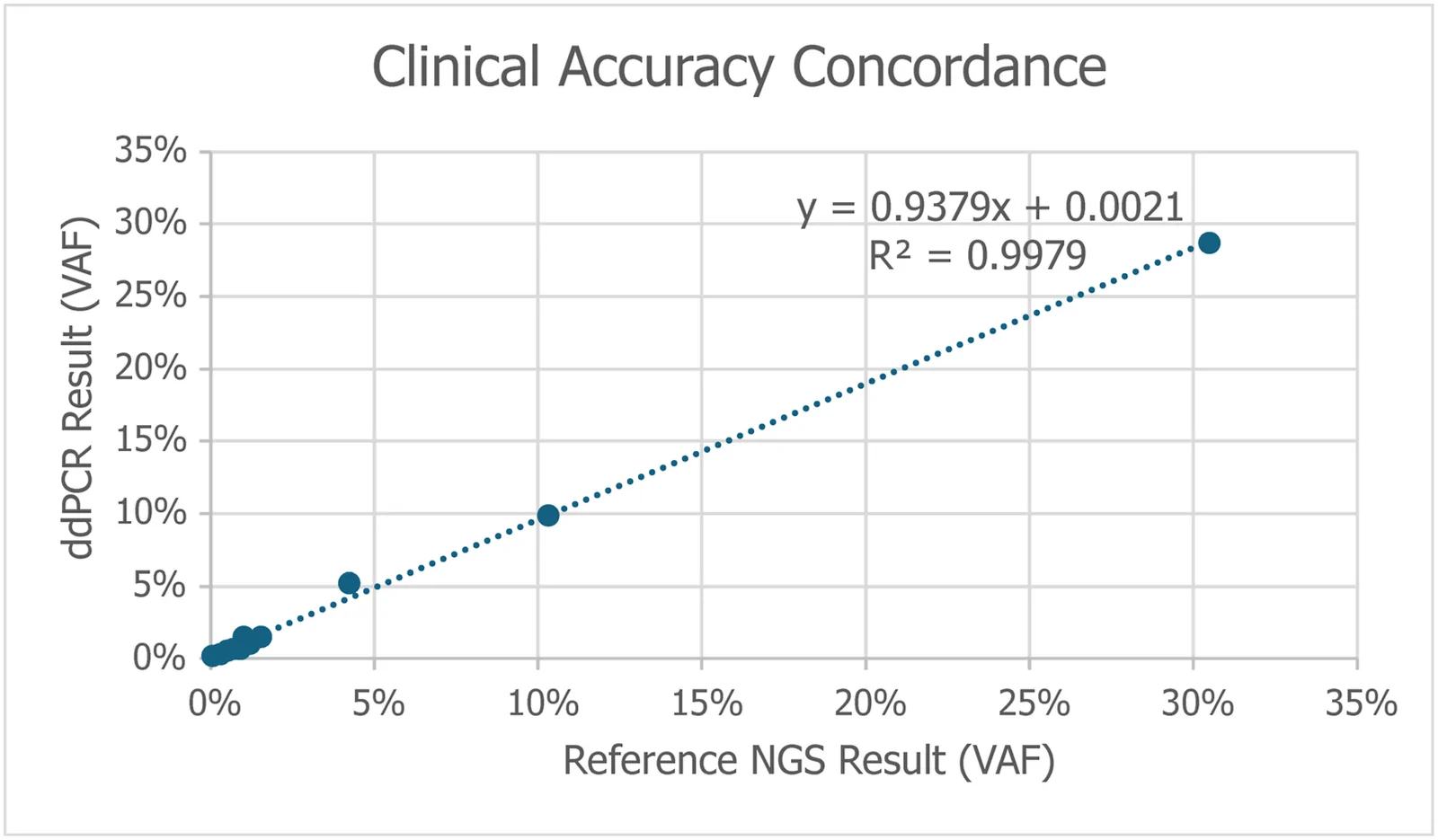
Clinical Accuracy Concordance. Linear regression analysis was performed to assess the concordance between the VAFs of the GeneStrat *ESR1* ddPCR Test and the reference NGS results for the 11 eligible *ESR1* reference variants. The equation for the best-fit trendline is provided, along with the coefficient of determination (R^2^) for the linear regression.

## 4 Discussion

We have developed and validated a clinical-grade, blood-based ddPCR test for the multiplexed detection of the 11 most prevalent and actionable variants in the ligand binding domain of *ESR1*. Leveraging the advanced multiplexing capabilities of the QX600 system, the 11 analytes are detected using as few as 6 reaction wells with cfDNA inputs ranging from 0.4 to 36 ng per well. This highly multiplexed test conformation allows for reduced subsampling and therefore higher input per well, potentially improving test sensitivity as compared to lower multiplexing systems. Moreover, the inclusion of the Automated Droplet Generator improves reproducibility by reducing hands-on time for specimen processing as compared to manual droplet generation.

Although intended for use as a qualitative assay to aid in clinical treatment decision-making with positive or negative results, the GeneStrat *ESR1* ddPCR Test may be utilized for semi-quantitative evaluation of clinical cutoffs in other settings such as research and clinical studies. To support such research studies, we assessed the lower LoQ and linearity for each individual analyte and found the test to be linear across the full range of inputs (∼10-1000 mutant copies/test) with an average LoQ of 67 mutant copies/test (IQR: 37-95 mutant copies/test).

The ddPCR test was validated for clinical accuracy using 25 HR+/HER2-advanced breast cancer (ABC) patient specimens and found to be 100% concordant when compared to the FDA-authorized Guardant360 or NY State CLEP-approved MSK-ACCESS clinical NGS tests (11/11 eligible *ESR1* variants and 18/18 *ESR1* WT samples were correctly called). The ddPCR test was also highly concordant with the reference test at the variant allele frequency level (R^2^ = 0.9979). These results were aligned with the results of our analytic studies. Analytic specificity was 100% based on initial testing using wild-type analytic materials as well as data generated using a cohort of ten normal healthy donors. The variant-level LoD95 was analytically demonstrated as low as 0.065% VAF and 7 mutant copies (maximum: 0.2% VAF, 22.9 mutant copies) using 30 contrived positive samples and was confirmed with our clinical accuracy cohort where reference variants were detected as low as 0.2% VAF and 8.4 mutant copies.

It is notable that one variant was detected by the GeneStrat *ESR1* ddPCR Test but not the reference NGS test. *ESR1* L536R was found at 0.01% VAF and 3 mutant copies in MSK-BDX-ESR1-037 by ddPCR but was not detected by the comparator method, Guardant360 (G360). This sample was positive for 3 other *ESR1* variants, thus the single variant discordance did not impact the positive label-level result for this subject nor the resulting accuracy calculation. We note that the maximum input to the G360 CDx is 30 ng cfDNA [28], and the ddPCR input for this specimen was approximately 100 ng, suggesting that the discordance may have resulted from differential input quantities. Since only 3 copies of the variant were detected in 100 ng, it may not have been detectable at the 30-ng input level with the NGS test. Moreover, the LoD95 for the G360 CDx test is 0.2-0.3% VAF at 30 ng input, which is above the 0.01% VAF at which this variant was detected by the ddPCR test.

Another difference between the approved G360 CDx (and MSK-ACCESS) and the newly developed ddPCR test is that the latter produces a non-discriminating readout for four of the 11 covered variants, V422del, L536H, L536P, and Y537D. Thus, specimens that are polyclonal for any of the above variants will not be distinguished within the ddPCR test from those that are positive for a single variant. Importantly, this is not viewed as a limitation in the context of current treatment guidance, which does not differentiate between these *ESR1* mutations.

The GeneStrat *ESR1* ddPCR Test provides discriminating outputs for the E380Q, L536R, S463P, Y537 C/N, as well as the D538G and Y537S variants which account for >70% of LBD resistance mutations. [10], [29]. In our clinical accuracy cohort of only n = 25 patients, we observed that the majority of reference variants (6 of 11; 55%) were either D538G or Y537S. This discriminating capability of the assay for these variants may provide future utility for treatment selection should the therapeutic landscape evolve to support *ESR1* variant-specific treatment decisions. For example, the PlasmaMATCH study assessed the relationship between *ESR1* mutations and response to fulvestrant in AI pretreated patients. The investigators found that progression free survival was longer in patients with a detectable baseline Y537C variant (and shorter with Y537S) as compared to other *ESR1* variants [30].

A limitation of targeted applications that are PCR based when compared to NGS is that coverage of the genome may be insufficient to support detection of all actionable variants in a test specimen. While *ESR1* activating mutations are currently one of the most prevalent known mechanisms of acquired resistance to ET in HR+/HER2- ABC, there are several other established resistance drivers, potentially justifying a more comprehensive approach to diagnostic testing [31], [32]. However, while NGS may provide the breadth of coverage needed to comprehensively identify multiple actionable genomic alterations in a diagnostic setting, technical variables such as highly complex (and non-standardized) wet lab and data analysis workflows can create hurdles to the generation of accurate and consistent results [33]. Amplicon-based sequencing panels can also produce data with low coverage uniformity due to the use of large primer pools; whereas PCR based assays are generally comprised of smaller multiplexes which can more easily be optimized for balanced assay efficiency. Additionally, the overall complexity of NGS data outputs, as well as the reporting of incidental findings and variants of unknown significance (VUS) can create barriers to NGS adoption by clinicians [34], [35]. Digital PCR methods, such as ddPCR utilized here, include a less complex wet lab workflow, and direct measurement of only the variants of interest. Finally, digital PCR methods may provide measurement of the actual number of analyte molecules present in the sample; while raw NGS data must be extensively manipulated using a series of bioinformatic tools to produce results, requiring significant investment in error correction tools and potential for variability across laboratories or runs.

In our view, digital PCR based assays may be most clinically relevant, given the ultra-high sensitivity required for the identification of emergent *ESR1* mutations in peripheral blood. Also worth considering is the effectiveness of ddPCR as a serial monitoring strategy as compared to NGS due to lower cost, reduced reimbursement complexity for monitoring applications, and significantly shorter turnaround time [26], [36]. Though the cost of NGS has dramatically reduced over the past decade [37], most highly adopted comprehensive genomic panels (CGPs) remain more expensive to run than ddPCR [36], and data management and storage requirements add a significant burden for the average diagnostic laboratory. Alternatively, PCR datafiles are typically less than a gigabyte in size and can be analyzed without specialized bioinformatic tools or highly trained analysts.

Ultimately, NGS and PCR represent complementary approaches to molecular diagnostic testing. NGS CGPs may be the more appropriate method when broad genomic profiling is required, especially when prior knowledge of the alterations of interest is lacking. On the other hand, ddPCR is most efficient for deeper and repeated interrogation of specific targets, especially in the context of surveillance for a small set of known mutations, rendering it an ideal application for the detection of the emergent 11 *ESR1* LBD mutations. In the context of endocrine resistant HR+/HER2-advanced breast cancer, as new therapies are approved for patients harboring one or more ET resistance mutations, additional ddPCR assays (such as *PIK3CA* mutation assays) can be run in combination with the *ESR1* ddPCR Test for a streamlined, precise testing strategy that is also highly informative for treatment guidance. Digital PCR can be integrated into a diagnostic algorithm for monitoring, in conjunction with an up-front NGS-based test. In the context of the recent approval of camizestrant, where the approved NGS test is indicated for use by the FDA, ddPCR could be used to further identify patients with emergent *ESR1* mutations with its ultrasensitive LoD or as a lower cost, rapid turnaround orthogonal confirmatory test when the NGS test is inconclusive.

## 5 Conclusions

We have developed and validated a clinical-grade, blood-based digital PCR laboratory-developed test, the GeneStrat *ESR1* ddPCR Test, for the ultrasensitive detection of prevalent *ESR1* ligand-binding domain mutations in patients with advanced HR+/HER2− breast cancer. The assay demonstrated highly sensitive mutation detection across the targeted variant panel, high specificity in wild-type specimens, reproducible qualitative performance, and strong concordance with orthogonal clinical ctDNA profiling.

These findings support the use of multiplex digital PCR as a rapid, sensitive, and scalable liquid biopsy approach for identifying emerging *ESR1* mutations and informing endocrine therapy selection in the evolving treatment landscape for HR+/HER2− advanced breast cancer. Future studies evaluating serial testing in larger, prospective cohorts may further define the clinical utility of ddPCR-based *ESR1* monitoring for earlier detection of endocrine resistance and molecularly guided treatment intervention.

## Author contributions

Conceptualization: GP, LJ, EL, HH, KE, EB, TE and PR; Methodology: LJ, GP, EL, KE, EB, TE, HH and PR; Resources: EB, TE, BD, GP, KS, EL, KE and PR; Investigation: HH, BD, EB, TE, KS, EL and KE; Data Curation: EB, TE, LJ, HH, KE, EL, KS, GP and PR; Formal Analysis: LJ, EB, TE, KS, HH, EL, GP, KE and PR; Writing – original draft: LJ, HH, BD, KE, GP, EL, EB, TE, PR; Writing – review and editing: HH, BD, EL, KE, EL, EB, TE, GP, PR.

## Ethics declaration

Written informed consent to take part in the study and to publish the article has been obtained from all participants or their legal representatives. The privacy rights of participants have been observed.

This study included organ or tissue donors. This study includes human biological material and consent was obtained by donors, or their next of kin or legal representatives, for use in this study and for publication of the article. The samples used in this research were not sourced from executed prisoners or prisoners of conscience.

This study was performed in compliance with relevant laws, regulatory frameworks and guidelines where the research took place. This study was approved by the Memorial Sloan Kettering Cancer Center. (Approval No. IRB #22-292)

## Funding sources

This research did not receive any specific grant from funding agencies in the public, commercial, or non-profit sectors.

## Declaration of competing interest

The authors declare that they have no known competing financial interests or personal relationships that could have appeared to influence the work reported in this paper.

## Figures and Tables

**Fig. S1:**
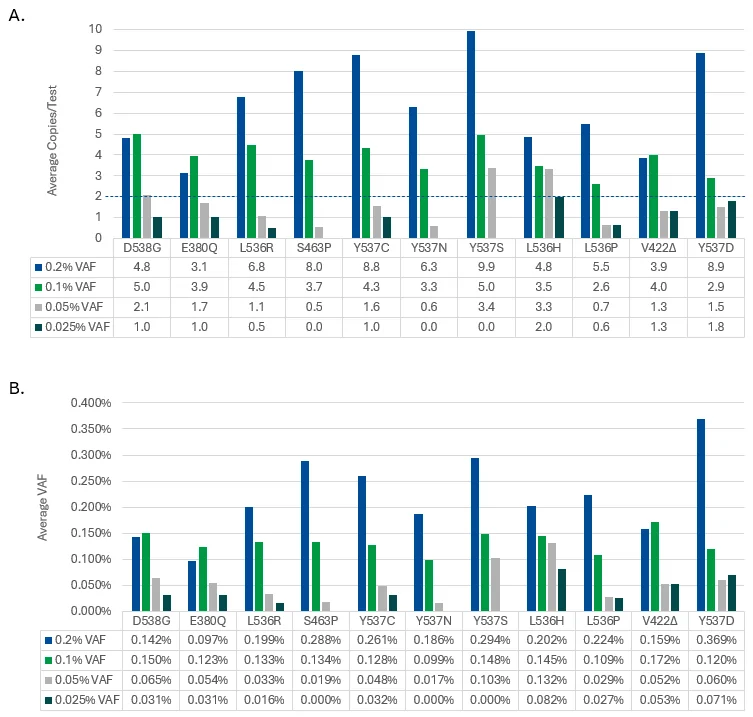
Feasibility with 10 ng input. The SeraSeq ctDNA ESR1 Mutation Mix AF1% was used for D538G, E380Q, L536R, S463P, and Y537 C/N/S to assess initial test accuracy. Contrived samples were generated using synthetic dsDNA for L536 H/P, V422del, and Y537D. Samples were tested at 0.2% - 0.025% VAF. The average observed copies per test and the average observed VAF for each concentration are shown in (A) and (B), respectively for the 10 ng input per reaction condition. The analytic threshold for calling a positive was set to 2 copies/test (dotted blue line in (A)).

**Fig. S2:**
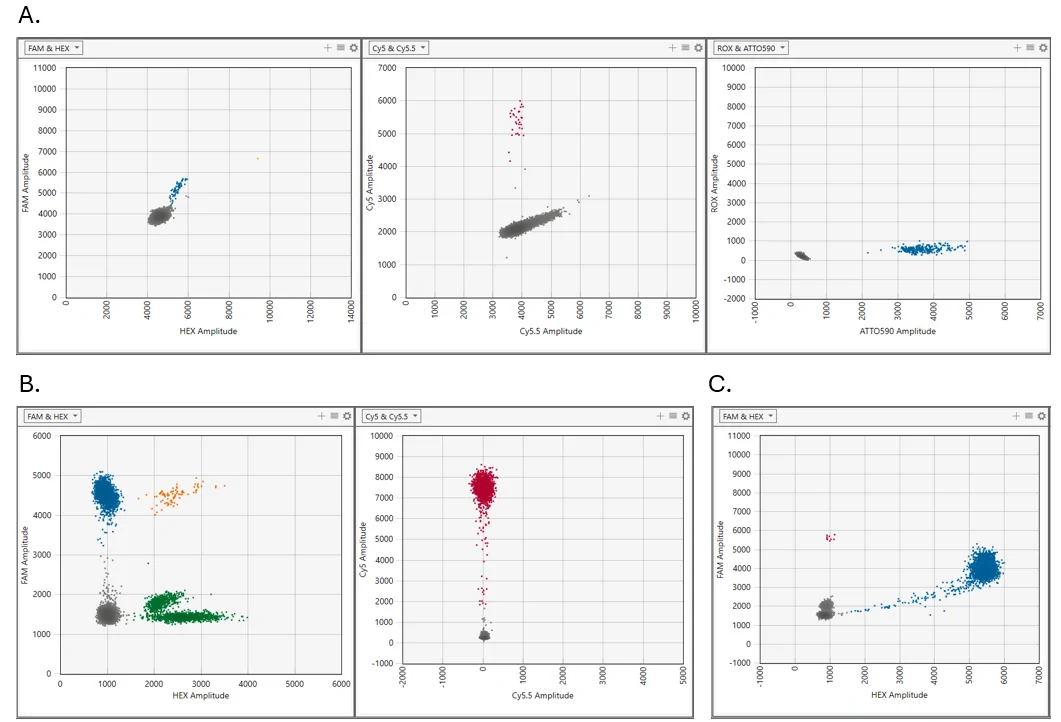
Clinical Accuracy Representative 2D Plots. (A) MSK-BDX-ESR1-011 tested positive with the ddPLEX assay for *ESR1* variants D538G (blue data points in FAM/HEX plot) and Y537N (red data points in Cy5/Cy5.5 plot). The internal ddPCR control signal is shown in blue in the far right, ROX/ATTO590 plot, and ddPLEX WT plots are shown in (B). (C) MSK-BDX-ESR1-002 tested positive for an EXV assay variant (red data points). The WT control signal is represented by blue data points.
