# Supplementary material for: Development of a clinical-grade ultrasensitive droplet digital PCR test for monitoring of relevant ESR1 variants by liquid biopsy
Source: J Liq Biopsy. 2026 Oct 13;14:100675. doi: 10.1016/j.jlb.2026.100675 (PMC13697279; doi:10.1016/j.jlb.2026.100675)
Supplement: Supplementary file 1 [file mmc1.docx]

**Supplementary Table S1**. Sequence information for the synthetic double stranded DNA (gBlocks, IDT) used to construct contrived positive samples for analytic validation studies

| gBlock | Sequence |
| --- | --- |
| *ESR1* D538G  (c.1613 A>G) | AGTCCTTTCTGTGTCTTCCCACCTACAGTAACAAAGGCATGGAGCATCTGTACAGCATGAAGTGCAAGAACGTGGTGCCCCTCTATGGCCTGCTGCTGGAGATGCTGGACGCCCACCGCCTACATGCGCCCACTAGCCGTGGAGGGGCATCCGTGGAGGAGAC |
| *ESR1* E380Q  (c.1138 G>C) | CATTTGAGTCAGCAGGGTTTTTCTTGCTTGTTTTCAGGCTTTGTGGATTTGACCCTCCATGATCAGGTCCACCTTCTACAATGTGCCTGGCTAGAGATCCTGATGATTGGTCTCGTCTGGCGCTCCATGGAGCACCCAGGGAAGCTACTG |
| *ESR1* L536R  (c.1607 T>G) | AGTCCTTTCTGTGTCTTCCCACCTACAGTAACAAAGGCATGGAGCATCTGTACAGCATGAAGTGCAAGAACGTGGTGCCCCGCTATGACCTGCTGCTGGAGATGCTGGACGCCCACCGCCTACATGCGCCCACTAGCCGTGGAGGGGCATCCGTGGAGGAGAC |
| *ESR1* S463P  (c.1387 T>C) | TCTGGGTCTCCTAGACCTCATCCTCTTTGAGCTTCTCTCTCTCACTCTCTCTCTGCGCATTCAGGAGTGTACACATTTCTGCCCAGCACCCTGAAGTCTCTGGAAGAGAAGGACCATATCCACCGAGTCCTGGACAAGATCACAGACACTTTGATCCACCTGA |
| *ESR1* Y537C  (c.1610 A>G) | AGTCCTTTCTGTGTCTTCCCACCTACAGTAACAAAGGCATGGAGCATCTGTACAGCATGAAGTGCAAGAACGTGGTGCCCCTCTGTGACCTGCTGCTGGAGATGCTGGACGCCCACCGCCTACATGCGCCCACTAGCCGTGGAGGGGCATCCGTGGAGGAGAC |
| *ESR1* Y537N  (c.1609 T>A) | AGTCCTTTCTGTGTCTTCCCACCTACAGTAACAAAGGCATGGAGCATCTGTACAGCATGAAGTGCAAGAACGTGGTGCCCCTCAATGACCTGCTGCTGGAGATGCTGGACGCCCACCGCCTACATGCGCCCACTAGCCGTGGAGGGGCATCCGTGGAGGAGAC |
| *ESR1* Y537S  (c.1610 A>C) | AGTCCTTTCTGTGTCTTCCCACCTACAGTAACAAAGGCATGGAGCATCTGTACAGCATGAAGTGCAAGAACGTGGTGCCCCTCTCTGACCTGCTGCTGGAGATGCTGGACGCCCACCGCCTACATGCGCCCACTAGCCGTGGAGGGGCATCCGTGGAGGAGAC |
| EXD *ESR1* 422del | ATTTATTTTTGCTATGTTTTCATAGGAACCAGGGAAAAT GTGTAGAGGGCATGGAGATCTTCGACATGCTGCTGGCTACATCATCTCGGTTCCGC ATGATGAATCTGCAGGGAGAGGAGTTTGTGTGCCT |
| EXD *ESR1* L536H | TTCTGTGTCTTCCCACCTACAGTAACAAAGGCATGGAGCATCTGTACAGCATGAAG TGCAAGAACGTGGTGCCCCACTATGACCTGCTGCTGGAGATGCTGGACGCCCACC GCCTACATGCGCCCACTAGCCGTGGAGGGGCATCCGTGG |
| EXD *ESR1* L536P | TTCTGTGTCTTCCCACCTACAGTAACAAAGGCATGGAGCATCTGTACAGCATGAAG TGCAAGAACGTGGTGCCCCCCTATGACCTGCTGCTGGAGATGCTGGACGCCCACC GCCTACATGCGCCCACTAGCCGTGGAGGGGCATCCGTGG |
| EXD *ESR1* Y537D | TTCTGTGTCTTCCCACCTACAGTAACAAAGGCATGGAGCATCTGTACAGCATGAAG TGCAAGAACGTGGTGCCCCTCGATGACCTGCTGCTGGAGATGCTGGACGCCCACC GCCTACATGCGCCCACTAGCCGTGGAGGGGCATCCGTGG |

**Supplementary Table S2.** In-line QC Materials

| Control | Source | Expected Result | Workflow steps controlled |
| --- | --- | --- | --- |
| ddPLEX *ESR1* Positive Control | ddPLEX kit (Bio-Rad) | Positive for *ESR1* E380Q, L536R, S463P, Y537C/N/S, D538G and *ESR1* wild-type (WT) | ddPCR |
| *ESR1* Extended Variant ddPCR Positive Control | Biodesix | Positive for V422del, L536H, L536P, Y537D and *ESR1* WT | ddPCR |
| *ESR1* Analytic Positive Control | Biodesix | Positive for all 11 *ESR1* variants and *ESR1* WT | Extraction through ddPCR |
| Negative Specimen Control | Biodesix | Negative for all 11 *ESR1* variants and positive for *ESR1* WT | Extraction through ddPCR |
| No Template Control (NTC; Nuclease-free water) | Biodesix | Negative for all 11 *ESR1* variants and *ESR1* WT | ddPCR |
| Internal Test Control | ddPLEX kit (Bio-Rad) | Positive (unique, non-human target) | ddPCR (overall performance) |

**Supplementary Table S3**. Feasibility Results for WT Inputs

| Target | 30 ng Input  (Mutant Copies/Test) | 30 ng Input  (WT Copies/Test) | 10 ng Input  (Mutant Copies/Test) | 10 ng Input  (WT Copies/Test) |
| --- | --- | --- | --- | --- |
| D538G | 0.0 | 10012 | 0.0 | 3109 |
| E380Q | 0.0 | 9192 | 0.0 | 2998 |
| L536R | 0.0 | 10012 | 0.0 | 3109 |
| S463P | 0.0 | 8053 | 0.0 | 2747 |
| Y537C | 0.0 | 10012 | 0.0 | 3109 |
| Y537N | 1.7 | 10012 | 1.7 | 3109 |
| Y537S | 0.0 | 10012 | 0.0 | 3109 |
| L536H/L536P/V422del/Y537D Multiplex | 0.0 | 7365 | 0.0 | 2378 |

**Supplementary Table S4.** LoQ and Linearity Results Summary. The average mutant copies per test measured at the LoQ for each *ESR1* variant is provided. The coefficient of determination (R^2^) for the linearity evaluation is also provided (input range: approximately 10-1000 mutant copies/test).

| Target | LoQ (Avg Mutant Copies/Test) | Linear Regression R^2^ |
| --- | --- | --- |
| D538G | 90 | 0.9999 |
| E380Q | 136 | 0.961 |
| L536H | 71 | 0.9995 |
| L536P | 17 | 0.9995 |
| L536R | 37 | 0.9949 |
| S463P | 25 | 0.999 |
| V422del | 40 | 0.9884 |
| Y537C | 126 | 0.9989 |
| Y537D | 60 | 0.9968 |
| Y537N | 36 | 0.9991 |
| Y537S | 100 | 0.9992 |

**Supplementary Table S5.** Test Reproducibility (Mutant Copy Number). The coefficient of variation (CV) is provided for three runs performed on 3 separate days (Inter-Day), 3 runs performed on the same day (Intra-Day), and 5 total runs (Overall) across 3 input concentrations.

| Input Target | Condition | D538G | E380Q | L536R | S463P | Y537C | Y537N | Y537S | L536H | L536P | Y537D | 422del |
| --- | --- | --- | --- | --- | --- | --- | --- | --- | --- | --- | --- | --- |
| 1000 Copies | Inter-Day | 5.70% | 3.00% | 4.50% | 4.40% | 3.10% | 1.70% | 1.40% | 4.20% | 4.30% | 3.40% | 10.50% |
|  | Intra-Day | 7.30% | 3.60% | 6.80% | 5.10% | 4.60% | 1.50% | 2.70% | 0.80% | 4.40% | 4.60% | 2.30% |
|  | Overall | 5.70% | 4.20% | 5.70% | 5.50% | 3.70% | 1.60% | 3.60% | 3.70% | 3.50% | 3.60% | 9.70% |
| 250 Copies | Inter-Day | 4.80% | 4.90% | 8.50% | 9.40% | 6.70% | 4.50% | 10.50% | 3.40% | 10.00% | 2.70% | 6.40% |
|  | Intra-Day | 4.30% | 5.70% | 1.80% | 5.90% | 6.40% | 6.50% | 3.80% | 4.10% | 11.70% | 5.80% | 7.10% |
|  | Overall | 4.90% | 5.10% | 6.80% | 9.00% | 5.80% | 5.50% | 8.00% | 4.80% | 9.90% | 4.40% | 6.00% |
| 20 Copies | Inter-Day | 28.10% | 30.30% | 49.50% | 13.40% | 44.60% | 18.40% | 37.50% | 0.70% | 29.40% | 13.00% | 32.00% |
|  | Intra-Day | 33.20% | 10.40% | 23.70% | 30.50% | 20.30% | 18.70% | 25.40% | 18.00% | 27.20% | 28.10% | 13.70% |
|  | Overall | 36.80% | 27.60% | 38.50% | 22.30% | 35.00% | 19.00% | 36.60% | 12.80% | 27.80% | 25.30% | 22.70% |

**Supplementary Table S6.** Test Reproducibility (Variant Allele Frequency). The coefficient of variation (CV) is provided for three runs performed on 3 separate days (Inter-Day), 3 runs performed on the same day (Intra-Day), and 5 total runs (Overall) across 3 input concentrations.

| Input Target | Condition | D538G | E380Q | L536R | S463P | Y537C | Y537N | Y537S | L536H | L536P | Y537D | 422del |
| --- | --- | --- | --- | --- | --- | --- | --- | --- | --- | --- | --- | --- |
| 11.00% | Inter-Day | 5.39% | 1.42% | 4.60% | 5.00% | 2.72% | 1.91% | 1.59% | 2.89% | 5.52% | 1.97% | 8.65% |
|  | Intra-Day | 9.57% | 3.74% | 6.68% | 6.47% | 7.30% | 4.35% | 4.49% | 1.75% | 4.24% | 2.97% | 2.49% |
|  | Overall | 7.08% | 3.78% | 5.75% | 5.91% | 5.33% | 3.36% | 4.53% | 2.43% | 4.05% | 2.63% | 7.88% |
| 2.70% | Inter-Day | 6.58% | 3.05% | 10.01% | 10.60% | 9.56% | 4.76% | 11.44% | 6.68% | 12.90% | 5.86% | 7.08% |
|  | Intra-Day | 6.02% | 4.67% | 3.62% | 4.63% | 9.30% | 7.26% | 6.29% | 6.40% | 12.06% | 4.24% | 7.15% |
|  | Overall | 5.90% | 4.07% | 7.43% | 9.73% | 7.89% | 6.11% | 9.09% | 6.62% | 11.16% | 4.88% | 5.93% |
| 0.22% | Inter-Day | 26.38% | 31.87% | 50.46% | 11.66% | 44.54% | 18.78% | 35.58% | 0.06% | 28.99% | 10.77% | 31.04% |
|  | Intra-Day | 29.02% | 6.10% | 29.03% | 37.28% | 16.97% | 25.54% | 20.27% | 19.92% | 28.08% | 28.19% | 11.02% |
|  | Overall | 35.32% | 30.09% | 37.45% | 27.90% | 34.28% | 22.05% | 35.31% | 14.02% | 27.76% | 24.26% | 21.90% |

**Supplementary Table S7.** Normal Healthy Donor (NHD) WT copy number results. Copy numbers are provided for the 3 ddPLEX WT alleles (380WT, 436WT, and 53XWT) and EXV WT for n=10 donor specimens and workflow controls.

| Sample | 380WT | 436WT | 53XWT | EXV WT |
| --- | --- | --- | --- | --- |
| ddPCR Positive Control | 9023 | 9585 | 9874 | 11417 |
| *ESR1* Analytic Positive Control | 11308 | 9741 | 12806 | 11677 |
| Negative Specimen Control | 605 | 655 | 656 | 642 |
| No Template Control | 1.2 | 0.0 | 1.2 | 0 |
| NHD1 | 312 | 416 | 478 | 398 |
| NHD2 | 1432 | 1420 | 1321 | 1317 |
| NHD3 | 506 | 534 | 535 | 523 |
| NHD4 | 2022 | 2455 | 2129 | 2059 |
| NHD5 | 655 | 693 | 601 | 645 |
| NHD6 | 1849 | 2000 | 1849 | 1881 |
| NHD7 | 2187 | 2040 | 2026 | 1867 |
| NHD8 | 1166 | 1080 | 1086 | 1139 |
| NHD9 | 341 | 338 | 377 | 323 |
| NHD10 | 1183 | 1167 | 1045 | 999 |

**Supplementary Table S8.** Clinical Accuracy Specimens. Asterisks indicate samples whose cfDNA concentrations were below the limit of detection of the Qubit assay.

| Specimen ID | Plasma Volume (mL) | cfDNA Yield (ng) | # Wells  (Per Assay) | Input  (ng/Well) | Input  (ng/Assay) |
| --- | --- | --- | --- | --- | --- |
| MSK-BDSX-ESR1-002 | 5 | 59 | 3 | 6.4 | 19 |
| MSK-BDSX-ESR1-003 | 3.5 | 72 | 3 | 7.9 | 24 |
| MSK-BDSX-ESR1-004 | 3 | 106 | 3 | 11.5 | 35 |
| MSK-BDSX-ESR1-006 | 2.2 | <2.5* | 3 | <0.27 | <0.82 |
| MSK-BDSX-ESR1-009 | 3.2 | 3.3 | 3 | 0.4 | 1.1 |
| MSK-BDSX-ESR1-010 | 3.2 | <2.5* | 3 | <0.27 | <0.82 |
| MSK-BDSX-ESR1-011 | 3.5 | 114 | 3 | 12.4 | 37 |
| MSK-BDSX-ESR1-012 | 3 | 59 | 3 | 6.4 | 19 |
| MSK-BDSX-ESR1-013 | 3.3 | 47 | 3 | 5.1 | 15 |
| MSK-BDSX-ESR1-014 | 3.2 | 440 | 11 | 15 | 165 |
| MSK-BDSX-ESR1-015 | 3.5 | 108 | 3 | 11.8 | 35 |
| MSK-BDSX-ESR1-017 | 3 | 80 | 3 | 8.7 | 26 |
| MSK-BDSX-ESR1-018 | 3.5 | 25 | 3 | 2.7 | 8 |
| MSK-BDSX-ESR1-019 | 3 | 43 | 3 | 4.7 | 14 |
| MSK-BDSX-ESR1-021 | 3.5 | 126 | 3 | 13.7 | 41 |
| MSK-BDSX-ESR1-022 | 3.5 | 41 | 3 | 4.5 | 14 |
| MSK-BDSX-ESR1-029 | 3.5 | 38 | 3 | 4.1 | 12 |
| MSK-BDSX-ESR1-031 | 3.5 | 131 | 3 | 14.3 | 43 |
| MSK-BDSX-ESR1-033 | 3.5 | 44 | 3 | 4.7 | 14 |
| MSK-BDSX-ESR1-034 | 3.5 | 25 | 3 | 2.7 | 8 |
| MSK-BDSX-ESR1-035 | 3.5 | 63 | 3 | 6.9 | 21 |
| MSK-BDSX-ESR1-037 | 3.5 | 328 | 3 | 35.8 | 107 |
| MSK-BDSX-ESR1-038 | 3.5 | 66 | 3 | 7.1 | 21 |
| MSK-BDSX-ESR1-039 | 3.5 | 202 | 4 | 15 | 60 |
| MSK-BDSX-ESR1-040 | 3.5 | 45 | 3 | 4.9 | 15 |

**Supplementary Table S9.** Clinical accuracy for the GeneStrat *ESR1* ddPCR test versus the reference NGS tests shown in a sample label-level confusion matrix.

|  | **Reference NGS Result** | | |
| --- | --- | --- | --- |
|  |  | Positive | Negative |
| **ddPCR** | Positive | 7 | 0 |
|  | Negative | 0 | 18 |
